# Association between urinary metals and prostate-specific antigen in aging population with depression: a cross-sectional study

**DOI:** 10.3389/fpubh.2024.1401072

**Published:** 2024-05-23

**Authors:** Liquan Ren, Yue Zhang, Jinyi Wu

**Affiliations:** ^1^Department of Public Health, Wuhan Fourth Hospital, Wuhan, China; ^2^School of Public Health, Shanxi Medical University, Taiyuan, China; ^3^School of Public Health, Fudan University, Shanghai, China

**Keywords:** urinary metals, prostate-specific antigen, depression, NHANES, older adults

## Abstract

**Objective:**

This study aims to investigate the impact of depression and urinary metals on Prostate-Specific Antigen (PSA).

**Methods:**

Analysis was conducted on 1901 samples collected from the National Health and Nutrition Examination Survey (NHANES) database between 2001 and 2010. Analytical methods included stepwise multiple linear regression (MLR) analysis of the overall population’s urinary metals and PSA relationship, analysis of urinary metals and PSA relationship in older adults and BMI subgroups, analysis of urinary metals and PSA relationship in the depressed population, and restricted cubic spline (RCS) analysis. A significance level of *p* < 0.05 was considered statistically significant.

**Results:**

In the stepwise multiple linear regression, beryllium (Be) showed a dose–response association with PSA (third quartile: β = 0.05, 95%CI (0.02, 0.09); fourth quartile: β = 0.07, 95%CI (0.02, 0.12), *p* trend = 0.048). Subgroup analysis indicated that in individuals aged >60, Be at Q4 level [β = 0.09, 95%CI (0.05, 0.21)] exhibited a dose–response correlation with PSA. In the population with 25 ≤ BMI < 30, Be might more significantly elevate PSA, with Q4 level having a pronounced impact on PSA levels [β = 0.03, 95%CI (0.02, 1.27)]. In the depressed population, urinary cadmium (Cd) levels showed a significant positive dose–response relationship, with Q4 level of Cd having the maximum impact on PSA [β = 0.3, 95%CI (0.09, 0.49)].

**Conclusion:**

Individuals exposed to beryllium (Be), especially the older adults and overweight, should monitor their PSA levels. In depressed patients, cadmium (Cd) levels may further elevate PSA levels, necessitating increased monitoring of PSA levels among males.

## Introduction

According to the Global Burden of Disease (GBD) study, prostate cancer (PCa) remains a significant global health burden, with increasing incidence, mortality, and death rates over the past three decades (1, 2). These trends may continue with population aging. Globally, from 1990 to 2019, the incidence, death cases, and death rates of prostate cancer increased by 116.11, 108.94, and 98.25%, respectively. Prostate-specific antigen (PSA) is a serine protease that plays a crucial role in seminal fluid liquefaction in the prostate epithelium and serves as a key tumor marker, especially considering the lack of alternative markers for PCa (3–5). Although serum PSA levels are not a strong indicator of PCa presence, with only a 24% positive predictive value, the academic community still recommends using serum PSA as a standard for PCa screening and monitoring, contributing positively to early detection and prevention of the disease (6–9).

In the field of environmental and occupational medicine, exposure to toxic and carcinogenic metals can be traced back to 2,400 years ago (10). Metals become persistent environmental pollutants due to their resistance to microbial degradation, unlike organic pollutants (11–13). After ingestion through food, water, and air, the human body metabolizes and eliminates heavy metals in various ways, primarily through urine. The concentration of heavy metals in urine provides an indication of the body’s metal levels after metabolism (14, 15). The International Association for Research on Cancer (IARC) has classified beryllium and beryllium-containing compounds as Group 1 carcinogens. Many studies using rat models have resulted in tumor formation after exposure to beryllium compounds. Some of these studies have relied on intraperitoneal, intrapleural, or intramuscular injections of Be solutions (16, 17). In recent years, the impact of heavy metal exposure on mental disorders has gained widespread attention, but its effects are underestimated (18–20). Heavy metal exposure can cause neuroinflammation, oxidative stress, hormonal fluctuations, and disruption of neurotransmitters such as dopamine and serotonin, all potential pathogenic mechanisms for depression (21, 22).

Currently, there is limited analysis of the impact of co-exposure to mental disorders and heavy metals on disease. Therefore, analyzing the correlation between PSA and urine metals in the population with depression is of significant importance. Previous research on this topic is scarce and lacks studies with large samples and long durations. Thus, we utilized data from the National Health and Nutrition Examination Survey (NHANES) dataset comprising 1901 samples to explore the direct correlation between PSA and urine metals in the population with depression.

## Methods

### Dataset

The data used in this study come from five consecutive National Health and Nutrition Examination Survey (NHANES) cycles conducted between 2001 and 2010. NHANES is a population-based survey representative of the entire United States, aiming to assess the health and nutritional status of adults and children^1^ (16, 23, 24). Initially, 27,584 samples were extracted from a ten-year period.

**Figure 1:**
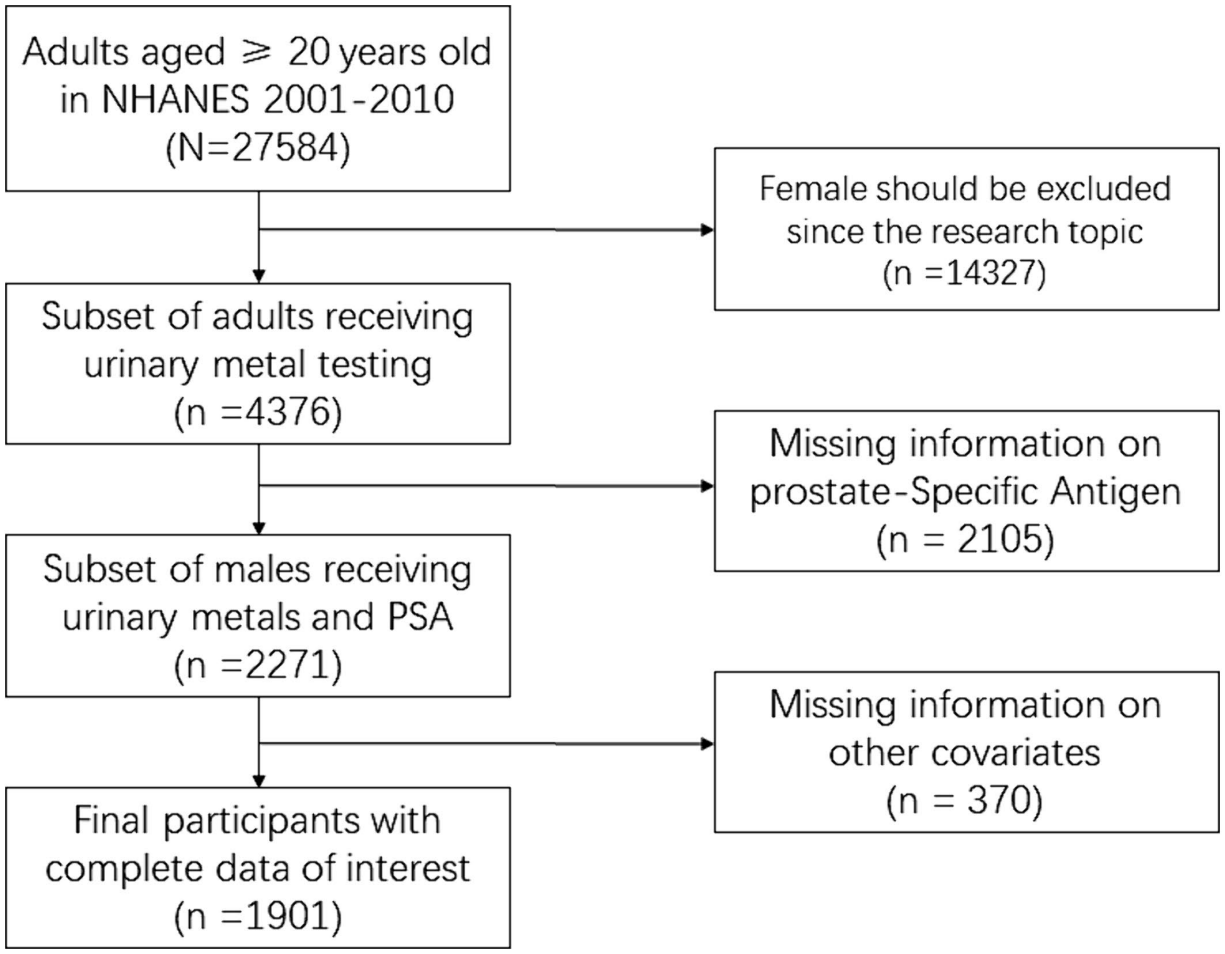
Flowchart of dataset selection.

To ensure relevance to prostate cancer, data from females (*n* = 14,327) were excluded. Subsequently, samples lacking PSA or confounding variable information were removed, further excluding 11,356 entries. Therefore, the final sample size of eligible adult males used in this study was *n* = 1,901. Detailed information on study design, sampling methods, inclusion, and exclusion criteria is available in Figure 1. Demographic data, physical exams, lab tests, and questionnaires were collected.

It was worth noting that this study specifically used publicly available data from NHANES, and ethical approval for this analysis was considered unnecessary (25).

### Assessment of urine metals

This study used urine metals as an indicator for exposure assessment, reflecting the accumulation of metals in the kidneys and other tissues. Analysis was performed using inductively coupled plasma mass spectrometry (ICP-MS), a multi-element technique. In this process, liquid samples were introduced into the ICP through a nebulizer and spray chamber, carried by flowing argon gas (26, 27). The resulting plasma was composed mainly of positive argon ions and electrons, which atomize and ionize the sample in the high-temperature region (6,000–8,000 K). The ions and argon gas were then introduced into the mass spectrometer, where the ICP operating at atmospheric pressure was separated from the mass spectrometer operating at 10–6 Torr. The mass spectrometer rapidly detected ions to determine the isotopic composition of elements. The electrical signals generated by ion detection were processed into digital information, representing ion intensity and subsequent element concentrations (16, 23). ICP-MS was used to determine the concentrations of seven elements in urine: barium (Ba), beryllium (Be), cobalt (Co), cesium (Cs), molybdenum (Mo), lead (Pb), antimony (Sb), and thallium (Tl). Urine samples were diluted 1 + 9 with 2% volume/volume double-distilled nitric acid (GFS Chemicals, Columbus, Ohio), which contained iridium and rhodium as multiple internal standards. Additionally, correction of urine cadmium (Cd) levels was performed using a formula to eliminate interference from molybdenum oxide: Corrected Cd level = Original Cd value − [(0.00175 * molybdenum) − 0.0136].

### PSA assessment

Total PSA results were obtained using the Hybritech method through Beckman Access. In this method, the second sample is introduced into an antibody container containing mouse monoclonal anti-PSA alkaline phosphatase conjugate and magnetic particles with a second mouse monoclonal anti-PSA antibody (24, 25). PSA in the sample binds to the immobilized monoclonal anti-PSA on the solid phase, while the anti-PSA conjugate reacts at a different antigenic site with PSA in the sample. The light produced in this reaction is proportional to the concentration of PSA in the sample.

### Assessment of depression

We used the Patient Health Questionnaire (PHQ-9), a depression screening tool consisting of ten items based on the Diagnostic and Statistical Manual of Mental Disorders IV (DSM-IV) criteria for depression diagnosis. It assesses the frequency of depressive symptoms over the past 2 weeks. Scores ranging from 0 to 3 for each symptom question represent “not at all,” “several days,” “more than half the days,” and “nearly every day,” respectively (23). The total score, calculated based on each participant’s answers to these 10 questions, serves as the standard score. Individuals with a total score equal to or exceeding 10 points were classified as patients with depression.

### Covariates

In-person interviews were conducted to collect data on various demographic factors, including age, gender, educational attainment, racial and ethnic classifications (Mexican American, Other Hispanic, Non-Hispanic White, Non-Hispanic Black, and others), family income-poverty ratios (PIR), smoking behaviors, and alcohol consumption. Physical examinations were used to determine the Body Mass Index (BMI), calculated by dividing weight (in kilograms) by the square of height (in meters).

The PIR was calculated by dividing family (or individual) income by the specific poverty guidelines for the survey year, accounting for household size and geographical location. A lower PIR indicated a higher level of poverty, while a ratio of one denoted equivalent income and poverty levels. Individuals were classified current smokers/past smokers/never smoke, and those who had consumed one or more alcoholic beverages in the past year were labeled as current drinkers.

Mobile centers were used for medical examinations, with body mass index (BMI) categorized as normal weight (<25), overweight (25 to <30), and obesity (≥30). Diabetes was defined as a fasting glucose level of ≥126 mg/dL or a reported prior diagnosis. Hypertension was defined as a persistent resting blood pressure (BP) of 140/90 mmHg or a reported prior diagnosis.

### Statistical analysis

#### Correlation analysis of PSA and urine metals

To investigate the correlation between PSA and nine urine metals comprehensively, we conducted a comprehensive analysis. First, we described demographic and medical history data, analyzing the geometric mean (GM), detection limit (LOD), and quartiles of the nine urine metals. Subsequently, we used analysis of variance and chi-square tests to assess differences in demographic data. Before regression, we conducted correlation matrix using Spearman method. Since our data was not normal distribution and there was no linear correlation, we chose Spearman correlation not Pearson correlation, which showed that variates were independent and suitable for multivariable linear regression (MLR).

#### Multivariable linear regression

In the second step, we performed MLR, gradually adjusting for demographic and medical history variables to determine the association between the natural logarithm-transformed PSA and quartiles of urine metals. MLR was conducted using the “survey” and “svydesign” commands in the R software package, considering weighting variables “WTSHM2YR” (cycles 2001–2002) and “WTSA2YR” (cycles 2003–2010), as well as stratum variable “SDMVSTRA”. Additionally, subgroup multivariable linear regression was conducted for PSA and significant urine metals.

#### Restricted cubic spline analysis

In the third step, we explored the dose–response relationship between PSA and significant urine metals using restricted cubic spline (RCS) analysis (22). All analyses were conducted using R software version 4.1.2 (R Foundation for Statistical Computing, Austria), with statistical significance set at two-sided *p* < 0.05.

## Results

### Participant characteristics

Quartiles of PSA were used to enumerate demographic and lifestyle characteristics (Table 1). With increasing PSA, age gradually increased (*p* = 0.001), PIR remained similar across different PSA levels (*p* = 0.755), the majority of the population was obese, and BMI varied across different PSA levels (*p* = 0.005). Non-Hispanic White individuals constituted the majority, and there were racial differences across different PSA levels (*p* = 0.007). Education level remained unchanged across different PSA levels (*p* = 0.221), the majority were married, and marital status varied across different PSA levels (*p* = 0.221). Non-alcohol drinkers were most common, with differences across different PSA levels (*p* = 0.395), and most individuals were current smokers, with differences across different PSA levels (*p* = 0.035); the majority did not have hypertension, with differences across different PSA levels (*p* = 0.643); and most individuals did not have diabetes, with differences across different PSA levels (*p* = 0.001). Depression did not differ significantly across different PSA levels (*p* = 0.178). Table 2 displays the geometric mean and four quartiles for the nine urine metals in our study. The records for all nine urine metals are reported in ng/mL. The correlation matrix showed that each covariate had no correlation with PSA, which demonstrated that included covariates were independent (Supplementary Figure S3).

**Table 1:** Baseline information in PSA quartiles of the NHANES 2001–2010.

PSA	Q1 (*N* = 475)	Q2 (*N* = 474)	Q3 (*N* = 470)	Q4 (*N* = 482)	F or Chi-squared value
**Age**					0.001*
	56.1 (11.7)	54.7 (11.6)	60.0 (12.4)	67.2 (11.5)	
**PIR**					0.755
	2.84 (1.64)	2.84 (1.61)	2.75 (1.60)	2.78 (1.58)	
**BMI**					0.005*
Normal (25<)	88 (18.53%)	111 (23.42%)	127 (27.02%)	131 (27.18%)	
Overweight (25 ≤ BMI < 30)	185 (38.95%)	175 (36.92%)	146 (31.06%)	144 (29.88%)	
obesity(≥30)	202 (42.53%)	188 (39.66%)	197 (41.91%)	207 (42.95%)	
**Race**					0.007*
Mexican American	77 (16.21%)	108 (22.78%)	103 (21.91%)	61 (12.66%)	
Other Hispanic	31 (6.53%)	31 (6.54%)	26 (5.53%)	24 (4.98%)	
Non-Hispanic White	277 (58.32%)	253 (53.38%)	245 (52.13%)	289 (59.96%)	
Non-Hispanic Black	76 (16%)	72 (15.19%)	81 (17.23%)	93 (19.29%)	
Other Race	14 (2.95%)	9 (1.9%)	15 (3.19%)	15 (3.11%)	
**Education**					0.221
Less than 9th grade	68 (14.32%)	67 (14.14%)	86 (18.3%)	88 (18.26%)	
9–11th grade	82 (17.26%)	77 (16.24%)	71 (15.11%)	61 (12.66%)	
High School	120 (25.26%)	112 (23.63%)	106 (22.55%)	115 (23.86%)	
Some College	113 (23.79%)	136 (28.69%)	106 (22.55%)	119 (24.69%)	
College Graduate or above	93 (19.58%)	82 (17.3%)	101 (21.49%)	99 (20.54%)	
**Marital status**					0.001*
Married	330 (69.47%)	327 (68.99%)	327 (69.57%)	328 (68.05%)	
Widowed	20 (4.21%)	22 (4.64%)	36 (7.66%)	55 (11.41%)	
Divorced	55 (11.58%)	44 (9.28%)	43 (9.15%)	49 (10.17%)	
Separated	10 (2.11%)	15 (3.16%)	13 (2.77%)	13 (2.7%)	
Never married	32 (6.74%)	30 (6.33%)	33 (7.02%)	21 (4.36%)	
Living with partner	29 (6.11%)	36 (7.59%)	18 (3.83%)	15 (3.11%)	
Refused	0 (0%)	0 (0%)	0 (0%)	1 (0.21%)	
**Alcohol**					0.395
Yes	138 (29.05%)	124 (26.16%)	130 (27.66%)	116 (24.07%)	
No	338 (71.16%)	350 (73.84%)	340 (72.34%)	366 (75.93%)	
**Smoking**					0.035*
Current smokers	210 (44.21%)	203 (42.83%)	222 (47.23%)	226 (46.89%)	
Past smokers	130 (27.37%)	123 (25.95%)	126 (26.81%)	119 (24.69%)	
Never smoke	135 (28.42%)	148 (31.22%)	122 (25.96%)	137 (28.42%)	
**Hypertension**					0.643
Yes	23 (4.84%)	16 (3.38%)	20 (4.26%)	23 (4.77%)	
No	453 (95.37%)	458 (96.62%)	150 (31.91%)	459 (95.23%)	
**Diabetes**					
Yes	78 (16.42%)	46 (9.7%)	77 (16.38%)	63 (13.07%)	0.001*
No	386 (81.26%)	419 (88.4%)	378 (80.43%)	406 (84.23%)	
Borderline	12 (2.53%)	9 (1.9%)	15 (3.19%)	13 (2.7%)	
**Depression**					0.038*
Yes	26 (5.47%)	32 (6.75%)	38 (8.09%)	42 (8.71%)	
No	449 (94.53%)	442 (93.25%)	432 (91.91%)	440 (91.29%)	

* *p* < 0.05; PSA, prostate-specific antigen; PIR, poverty income ratio; BMI, body mass index.

**Table 2:** Urine metals (ng/mL) in of NHANES 2001–2010.

Overall (*N* = 2,420)	Geometric mean	Q1	Q2	Q3	Q4
Barium	1.27	≤0.08	0.08–0.64	0.64–1.33	≥2.52
Beryllium	0.06	≤0.05	0.05–0.051	0.051–0.051	≥0.08
Cadmium	0.36	≤0.013	0.013–0.201	0.201–0.371	≥0.67
Cobalt	0.33	≤0.029	0.029–0.219	0.219–0.336	≥0.482
Cesium	4.79	≤0.191	0.191–3.26	3.26–5.08	≥7.48
Molybdenum	43.13	≤0.65	0.65–25.9	25.9–45.7	≥76.2
Lead	0.83	≤0.07	0.07–0.5	0.5–0.85	≥1.4
Antimony	0.08	≤0.023	0.023–0.049	0.049–0.075	≥0.13
Thallium	0.16	≤0.01	0.01–0.099	0.099–0.16	≥0.24

### Association of urine metal metabolites with PSA

**Figure 2:**
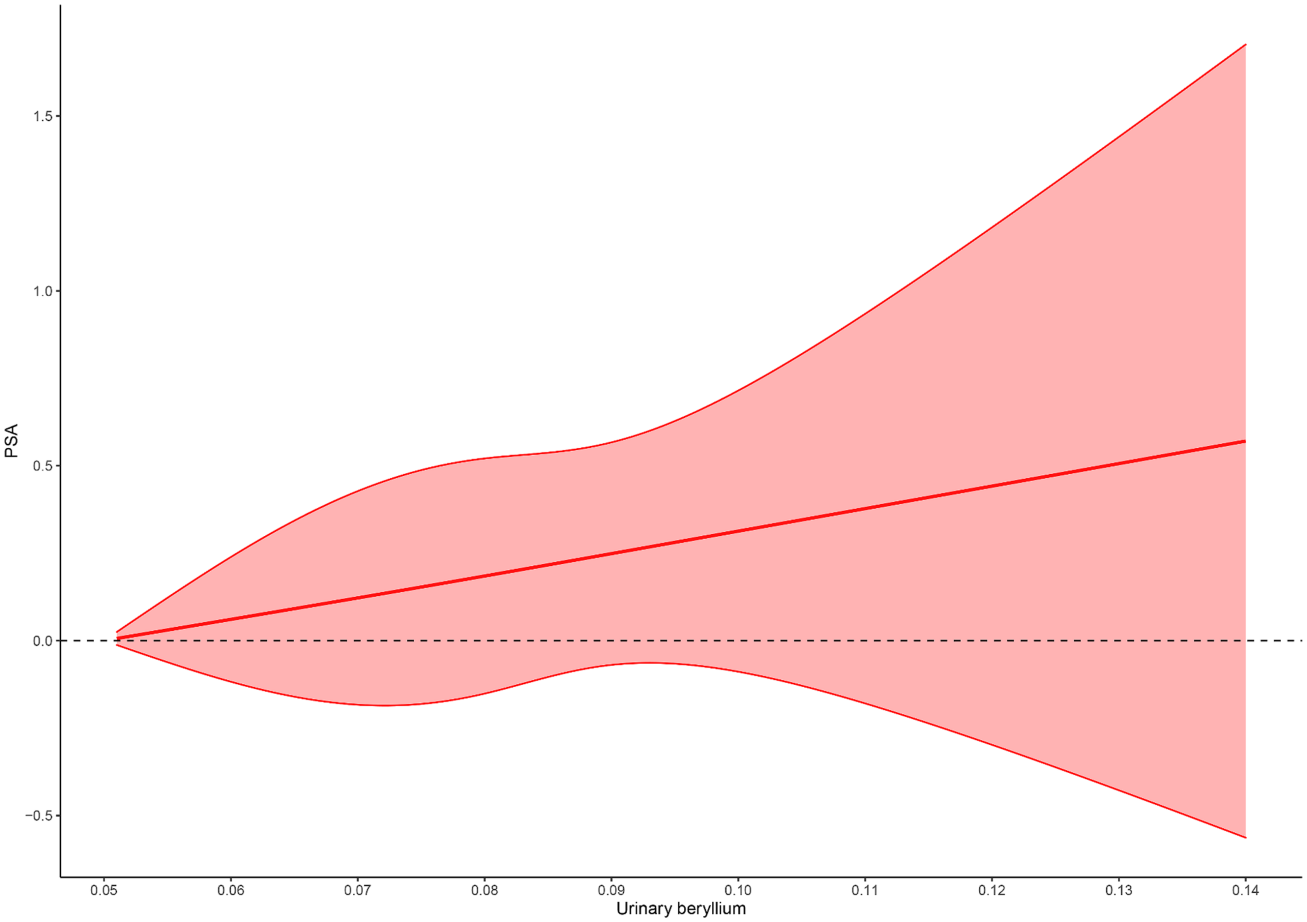
Restricted cubic spline for association between Be concentration with PSA. Adjusted age, gender, race, educational levels, BMI, family income-poverty ratio level, married status, smoking status, drinking status, diabetes and hypertension.

Table 3 shows the percentage differences in urine metals with PSA, using the first quartile as the reference. Be′s third and fourth quartiles [β = 0.05, 95%CI(0.02, 0.09); β = 0.07, 95%CI(0.02, 0.12), respectively] exhibited a trend of positive correlation with increasing PSA (trend *p* = 0.048). The fourth quartile associated with Cd was positively correlated with PSA increase [β = 0.2, 95%CI(0.01, 0.19)]. The fourth quartile associated with Co was also positively correlated with PSA increase [β = 0.05, 95%CI(0.01, 0.09)]. At the same time, urine Be had a dose–response association with PSA (Figure 2).

**Table 3:** Association between prostate-specific antigen and urine metals of NHANES 2001–2010.

	Model 1 β (95%CI)	Model 2 β (95%CI)	Model 3 β (95%CI)
**Urinary barium**	Reference	Reference	Reference
Q2	0.02 (−0.05, 0.09)	0.03 (−0.04, 0.09)	0.02 (−0.04, 0.08)
Q3	−0.01 (−0.08, 0.07)	0.03 (−0.04, 0.1)	0.02 (−0.05, 0.09)
Q4	−0.01 (−0.09, 0.06)	0.01 (−0.05, 0.08)	0.02 (−0.05, 0.08)
***p* trend**	0.54	0.75	0.70
**Urinary beryllium**	Reference	Reference	Reference
Q2	0.38 (0.27, 0.49)*	0.29 (0.18, 0.39)*	0.04 (0.02, 0.08)*
Q3	0.37 (0.26, 0.48)*	0.28 (0.17, 0.41)*	0.05 (0.02, 0.09)*
Q4	0.39 (0.21, 0.49)*	0.31 (0.12, 0.43)*	0.07 (0.03, 0.12)*
***p* trend**	0.035*	0.045*	0.048*
**Urinary cadmium**	Reference	Reference	Reference
Q2	0.01 (−0.06, 0.08)	−0.04 (−0.1, 0.04)	−0.04 (−0.1, 0.03)
Q3	0.07 (0, 0.16)*	−0.01 (−0.09, 0.07)	−0.02 (−0.1, 0.06)
Q4	0.2 (0.01, 0.19)*	−0.04 (−0.11, 0.05)	−0.05 (−0.13, 0.04)
***p* trend**	0.00*	0.51	0.39
**Urinary cobalt**	Reference	Reference	Reference
Q2	0.02 (−0.06, 0.08)	0.03 (−0.04, 0.09)	−0.01 (−0.08, 0.06)
Q3	0.04 (−0.04, 0.11)	0.03 (−0.04, 0.11)	0.02 (−0.05, 0.09)
Q4	0.06 (−0.03, 0.13)	0.05 (0.01, 0.09)*	−0.01 (−0.08, 0.07)
***p* trend**	0.15	0.93	0.98
**Urinary cesium**	Reference	Reference	Reference
Q2	0.07 (0, 0.15)	0.04 (−0.03, 0.1)	0.03 (−0.03, 0.1)
Q3	0.07 (−0.01, 0.15)	0.06 (−0.02, 0.13)	0.04 (−0.03, 0.11)
Q4	−0.01 (−0.09, 0.07)	−0.02 (−0.1, 0.06)	−0.04 (−0.12, 0.04)
***p* trend**	0.63	0.57	0.32
**Urinary molybdenum**	Reference	Reference	Reference
Q2	0.07 (−0.01, 0.14)	−2.92 (−25.75, 26.92)	−6.99 (−29.66, 22.99)
Q3	0.02 (−0.05, 0.09)	−6.59 (−29.67, 24.07)	−11.16 (−33.26, 18.25)
Q4	0.05 (−0.03, 0.14)	−18.62 (−39.29, 9.1)	−24.5 (−44.84, 3.33)
***p* trend**	0.39	0.93	0.87
**Urinary lead**	Reference	Reference	Reference
Q2	0.03 (−0.04, 0.1)	0.01 (−0.05, 0.08)	0 (−0.06, 0.07)
Q3	0 (−0.08, 0.08)	−0.04 (−0.1, 0.03)	−0.04 (−0.11, 0.03)
Q4	0.03 (−0.05, 0.11)	−0.06 (−0.13, 0.02)	−0.06 (−0.14, 0.02)
***p* trend**	0.73	0.07	0.06
**Urinary antimony**	Reference	Reference	Reference
Q2	0 (−0.07, 0.08)	−0.01 (−0.08, 0.05)	−0.01 (−0.07, 0.05)
Q3	−0.02 (−0.09, 0.06)	−0.02 (−0.09, 0.05)	−0.01 (−0.08, 0.06)
Q4	−0.08 (−0.16, 0)	−0.06 (−0.13, 0.01)	−0.06 (−0.13, 0.01)
***p* trend**	0.04	0.09	0.10
**Urinary thallium**	Reference	Reference	Reference
Q2	0.02 (−0.06, 0.1)	0.01 (−0.05, 0.08)	0.01 (−0.06, 0.07)
Q3	−0.01 (−0.09, 0.08)	0 (−0.07, 0.08)	−0.01 (−0.08, 0.07)
Q4	−0.06 (−0.15, 0.02)	0 (−0.08, 0.08)	−0.02 (−0.1, 0.06)
***p* trend**	0.09	0.93	0.58

^*^ *p* < 0.05. Model 1 = adjusted for urine creatinine; Model 2 = as Model 1 plus adjusted for sex, age (years, continuous), age squared, education (less than high school, high school graduate, some college and above), race (non-Hispanic white, non-Hispanic black, Mexican American, other), self-reported alcohol status (Yes and No) and self-reported smoking status (Current, Past and Never); Model 3 = Model 2 plus adjusted for BMI, self-reported hypertension (Yes and No) and self-reported diabetes (Yes and No).

### Age and BMI subgroup analysis

Subgroup analyses based on age and BMI were conducted for Be and PSA among depression patients. Clearly, Be significantly influenced PSA in the older adults subgroup (Supplementary Table S1). A dose–response relationship was observed between Be and PSA, with the highest effect in the Q4 level for individuals aged ≥60 [β = 0.09, 95%CI(0.05, 0.21)]. In the BMI group of 25 ≤ BMI < 30, the Q4 level significantly affected PSA levels [β = 0.03, 95%CI(0.02,1.27)], especially in individuals aged ≥60. In the 25 ≤ BMI < 30 group, Be may also more significantly increase PSA levels.

### Impact of heavy metals on PSA in depressed patients

In the population with depression, we observed an interesting phenomenon where urine cadmium (Cd) levels exhibited a clear positive dose–response relationship with PSA (Supplementary Figure S1). In model 3, the Q4 level of Cd had the greatest impact on PSA [β = 0.3, 95%CI(0.09, 0.49)], while beryllium (Be) showed no correlation in this population (Table 4). In the non-depressed population, the Q3 level of urine cobalt (Co) might increase PSA levels (Supplementary Table S2) [β = 0.29, 95%CI(0.16, 0.44)], without dose–response relationship (Supplementary Figure S2).

**Table 4:** Association between prostate-specific antigen and urine metals among depression participants.

	Model 1 β (95%CI)	Model 2 β (95%CI)	Model 3 β (95%CI)
**Urinary barium**	Reference	Reference	Reference
Q2	0.11 (−0.07, 0.29)	0.03 (−0.13, 0.18)	0.03 (−0.12, 0.18)
Q3	−0.06 (−0.21, 0.09)	−0.03 (−0.17, 0.12)	−0.02 (−0.17, 0.12)
Q4	0.08 (−0.09, 0.26)	0.04 (−0.13, 0.22)	0.05 (−0.13, 0.22)
***p* trend**	0.70	0.74	0.74
**Urinary beryllium**	Reference	Reference	Reference
Q2	0.07 (−0.07, 0.21)	0.08 (−0.04, 0.21)	0.03 (−0.12, 0.18)
Q3	0 (0, 0)	0 (0, 0)	−0.02 (−0.17, 0.12)
Q4	0 (0, 0)	0 (0, 0)	0.05 (−0.13, 0.22)
***p* trend**	0.36	0.18	0.22
**Urinary cadmium**	Reference	Reference	Reference
Q2	−0.1 (−0.29, 0.1)	0.14 (−0.32, 0.05)	0.2 (0.02, 0.39)*
Q3	−0.01 (−0.22, 0.2)	0.21 (0.01, 0.41)*	0.29 (0.07, 0.52)*
Q4	0.01 (−0.17, 0.2)	0.18 (0.1, 0.37)*	0.3 (0.09, 0.49)*
***p* trend**	0.56	0.04*	0.01*
**Urinary cobalt**	Reference	Reference	Reference
Q2	−0.11 (−0.29, 0.07)	0.08 (−0.04, 0.21)	−0.1 (−0.27, 0.07)
Q3	0.07 (−0.14, 0.28)	0 (0, 0)	0.06 (−0.14, 0.26)
Q4	0.02 (−0.16, 0.21)	0 (0, 0)	−0.06 (−0.24, 0.11)
***p* trend**	0.30	0.90	0.96
**Urinary cesium**	Reference	Reference	Reference
Q2	0.1 (−0.1, 0.31)	−0.02 (−0.2, 0.16)	−0.03 (−0.22, 0.16)
Q3	0.03 (−0.2, 0.25)	−0.05 (−0.26, 0.15)	−0.06 (−0.26, 0.15)
Q4	0.1 (−0.11, 0.32)	0.05 (−0.14, 0.25)	0.03 (−0.16, 0.23)
***p* trend**	0.49	0.64	0.79
**Urinary molybdenum**	Reference	Reference	Reference
Q2	−0.14 (−0.34, 0.06)	−0.27 (−0.46, −0.08)	−0.27 (−0.46, −0.08)
Q3	−0.13 (−0.29, 0.03)	−0.2 (−0.36, −0.03)	−0.2 (−0.36, −0.04)
Q4	0.01 (−0.21, 0.24)	−0.01 (−0.21, 0.19)	−0.01 (−0.21, 0.18)
***p* trend**	0.80	0.76	0.80
**Urinary lead**	Reference	Reference	Reference
Q2	0.05 (−0.14, 0.24)	0.01 (−0.17, 0.18)	0 (−0.18, 0.18)
Q3	−0.05 (−0.26, 0.17)	−0.09 (−0.28, 0.11)	−0.08 (−0.28, 0.12)
Q4	0.06 (−0.13, 0.25)	−0.02 (−0.19, 0.15)	−0.01 (−0.18, 0.16)
***p* trend**	0.84	0.55	0.68
**Urinary antimony**	Reference	Reference	Reference
Q2	0.1 (−0.09, 0.29)	0.04 (−0.13, 0.21)	0.03 (−0.15, 0.21)
Q3	0.16 (−0.03, 0.34)	0.09 (−0.11, 0.28)	0.08 (−0.11, 0.28)
Q4	0.03 (−0.15, 0.21)	0.04 (−0.13, 0.21)	0.04 (−0.13, 0.21)
***p* trend**	0.72	0.60	0.56
**Urinary thallium**	Reference	Reference	Reference
Q2	−0.05 (−0.25, 0.15)	−0.09 (−0.28, 0.09)	−0.1 (−0.28, 0.09)
Q3	−0.08 (−0.3, 0.14)	−0.07 (−0.27, 0.13)	−0.06 (−0.27, 0.14)
Q4	−0.16 (−0.38, 0.05)	−0.12 (−0.33, 0.09)	−0.13 (−0.35, 0.09)
***p* trend**	0.12	0.31	0.31

^*^ *p* < 0.05. Model 1 = adjusted for urine creatinine; Model 2 = as Model 1 plus adjusted for sex, age (years, continuous), age squared, education (less than high school, high school graduate, some college and above), race (non-Hispanic white, non-Hispanic black, Mexican American, other), self-reported alcohol status (Yes and No) and self-reported smoking status (Current, Past and Never); Model 3 = Model 2 plus adjusted for BMI, self-reported hypertension (Yes and No) and self-reported diabetes (Yes and No).

## Discussion

In this study, we conducted an in-depth analysis of the correlation between nine urine metals and PSA levels, finding a significant association, primarily with Be in males. Specifically, Be was found to potentially increase PSA levels. Beryllium is a natural element in the Earth’s crust and a byproduct in various manufacturing and energy production industries. Its sources include natural processes, such as rock weathering leading to its presence in water and soil, as well as anthropogenic activities involving its use in various manufacturing industries. Studies suggest that after exposure to Be, particularly due to the ionization of soluble effects of Be salts in aqueous solutions rather than the uptake of metal particles, there may be differential responses in renal tubular cells sensitive to Be, related to differences in PSA levels (28–30). Animal experiments have shown that Be can increase PSA production by directly affecting prostate cells (31–33). Be may also indirectly affect PSA levels by inhibiting PSA clearance. Metabolites of Be can be detected in urine, and this may serve as a biomarker for Be exposure.

Through dose–response relationship analysis, we found a significant dose–response relationship between Be and PSA, especially in the age group >60. This suggests that the impact of Be on PSA levels may vary with age, possibly due to increased sensitivity of older individuals to metals or cumulative effects of Be exposure in the older adults. In the group with 25 ≤ BMI < 30, increased sensitivity to metals may also be more likely, leading to an increase in PSA levels.

In recent years, mental health has become a noteworthy issue after a major epidemic event, and individuals with depression may be at a higher risk of developing health problems (34, 35). Numerous studies currently indicate that heavy metals, especially cadmium, can cause various abnormalities in aspects such as emotions, cognition, and behavior. Cadmium is not an essential metal element for the human body. After entering the body, cadmium has strong toxicity, accumulates continuously in the body, and causes damage to multiple organs (36–40). The slow metabolism and long half-life of cadmium in the body are the main reasons for chronic poisoning, leading to symptoms such as renal dysfunction, renal failure, liver and kidney damage, osteoporosis, and osteomalacia, as well as mental illnesses (41–44).

Our results show that an increase in urine cadmium levels enhances the increase in PSA levels in depressed patients. This indicates that depression and Cd may synergistically lead to an increase in PSA. Similar to the liver and kidneys, the prostate is considered a target organ for cadmium deposition. International Agency for Research on Cancer and epidemiological studies suggest that cadmium exposure may be a risk factor for prostate and kidney cancer (45–47). An *in vitro* study also suggests that cadmium can induce malignant prostate tumors (2, 8, 29). Chronic inflammation caused by beryllium is considered a possible contributor to the carcinogenicity of beryllium to humans (24, 48). Currently, there is no research analyzing the synergistic effects of Cd and depression on PSA, and in an era where mental health has a significant impact, further research on the mechanisms and preventive measures is warranted (12, 14, 42).

The findings of this study have important clinical and epidemiological significance. First, we provide new insights into the association between urine metals and PSA, particularly the impact of Be. Second, we found that the older adults population is more susceptible to the correlation between Be and PSA. Finally, in depression, Cd and depression may be synergistic risk factors for the increase in PSA. However, these findings still need further validation and a deeper understanding of the biological mechanisms between depression, urine metals, and prostate cancer.

This study has several limitations. Firstly, it exclusively involves American males, potentially reducing the study’s generalizability. Secondly, the data rely on participant recall, introducing the possibility of selection bias. Thirdly, the study employs a cross-sectional design rather than a cohort study, constraining causal analysis. Fourthly, the Spearman correlation insignificance might be influenced by noise or bias, while the further MLR had significant results.

## Conclusion

Overall, people exposed to beryllium (Be), particularly the older adults and those who are overweight, should regularly monitor their PSA levels. In individuals with depression, elevated cadmium (Cd) levels may also increase PSA levels, highlighting the importance of increased PSA monitoring in males.

## Data availability statement

The original contributions presented in the study are included in the article/Supplementary material, further inquiries can be directed to the corresponding author.

## Author contributions

LR: Formal analysis, Methodology, Writing – original draft. YZ: Investigation, Validation, Writing – original draft. JW: Conceptualization, Investigation, Methodology, Writing – review & editing.

## Footnotes

^1^https://www.cdc.gov/nchs/nhanes

## Supplementary material

SUPPLEMENTARY FIGURE S1 Restricted cubic spline for association between Be concentration with PSA among depression people. Adjusted age, gender, race, educational levels, BMI, family income-poverty ratio level, married status, smoking status, drinking status, diabetes and hypertension.

SUPPLEMENTARY FIGURE S2 Restricted cubic spline for association between Be concentration with PSA among non-depression people. Adjusted age, gender, race, educational levels, BMI, family income-poverty ratio level, married status, smoking status, drinking status, diabetes and hypertension.

SUPPLEMENTARY FIGURE S3 Spearman correlation matrix between included covariates and PSA.

## Glossary

PSC: Prostate carcinoma
PSA: Prostate specific antigen
NHANES: National Health and Nutrition Examination Survey
MLR: Multivariable linear regression
RCS: Restricted cubic spline
BMI: Body mass index
PIR: Poverty income ratio
Ba: Barium
Be: Beryllium
Cd: Cadmium
Co: Cobalt
Cs: Cesium
Mo: Molybdenum
Pb: Lead
Sb: Antimony
Tl: Thallium
CDC: Centers for Disease Control and Prevention
PCR: polymerase chain reaction
ICP-MS: Inductively coupled plasma mass spectrometry
GM: Geometric mean
CI: Confidential interval
IARC: International Association for research on cancer
OSHA: Occupational Safety and Health Administration
