# Supplementary figures and images for: Association between urinary metals and prostate-specific antigen in aging population with depression: a cross-sectional study
Source: Front Public Health. 2024 May 23;12:1401072. doi: 10.3389/fpubh.2024.1401072 (PMC11153824; doi:10.3389/fpubh.2024.1401072)

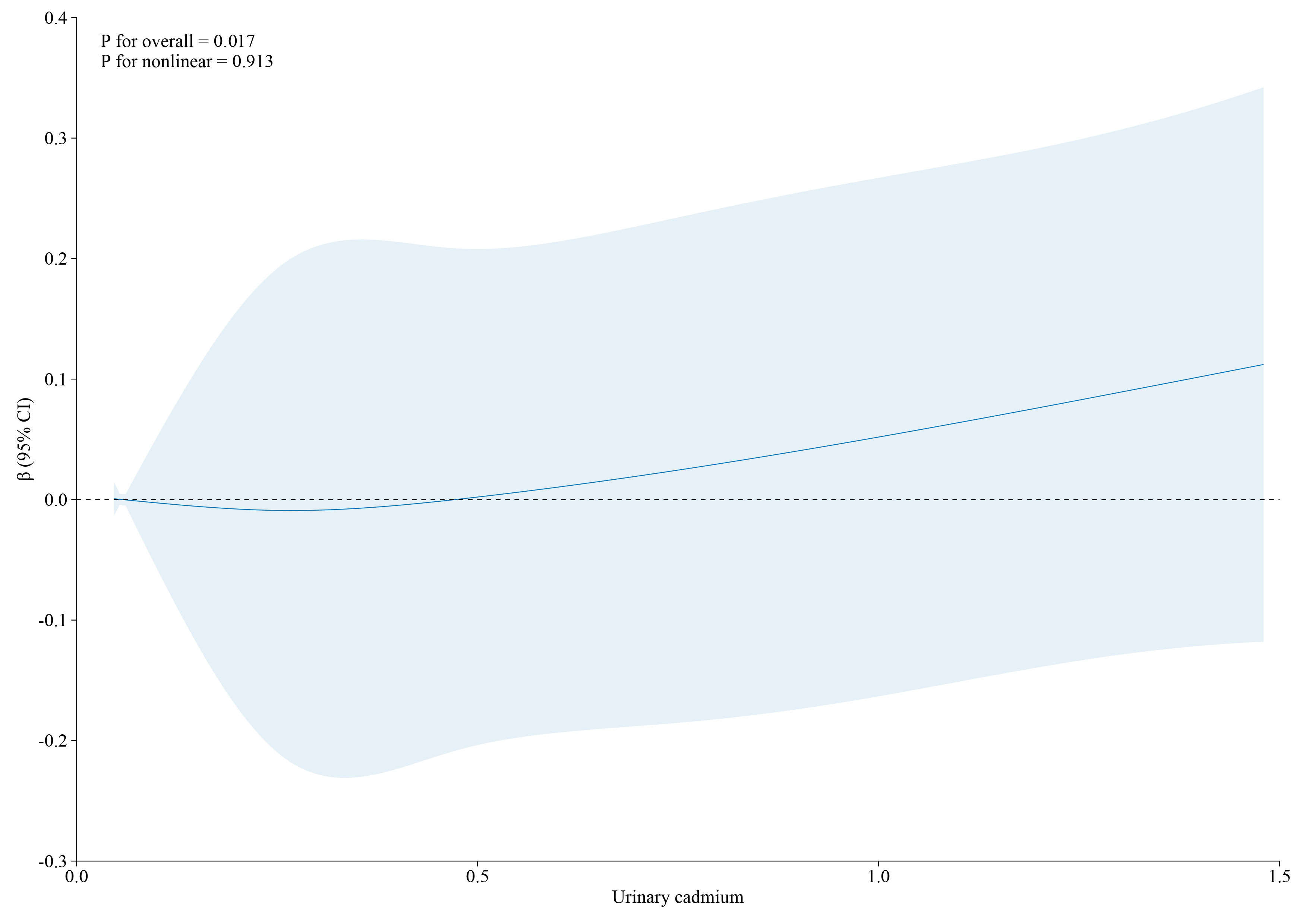

Supplement: SUPPLEMENTARY FIGURE S1 — Restricted cubic spline for association between Be concentration with PSA among depression people. Adjusted age, gender, race, educational levels, BMI, family income-poverty ratio level, married status, smoking status, drinking status, diabetes and hypertension. [file Image_1.TIF]

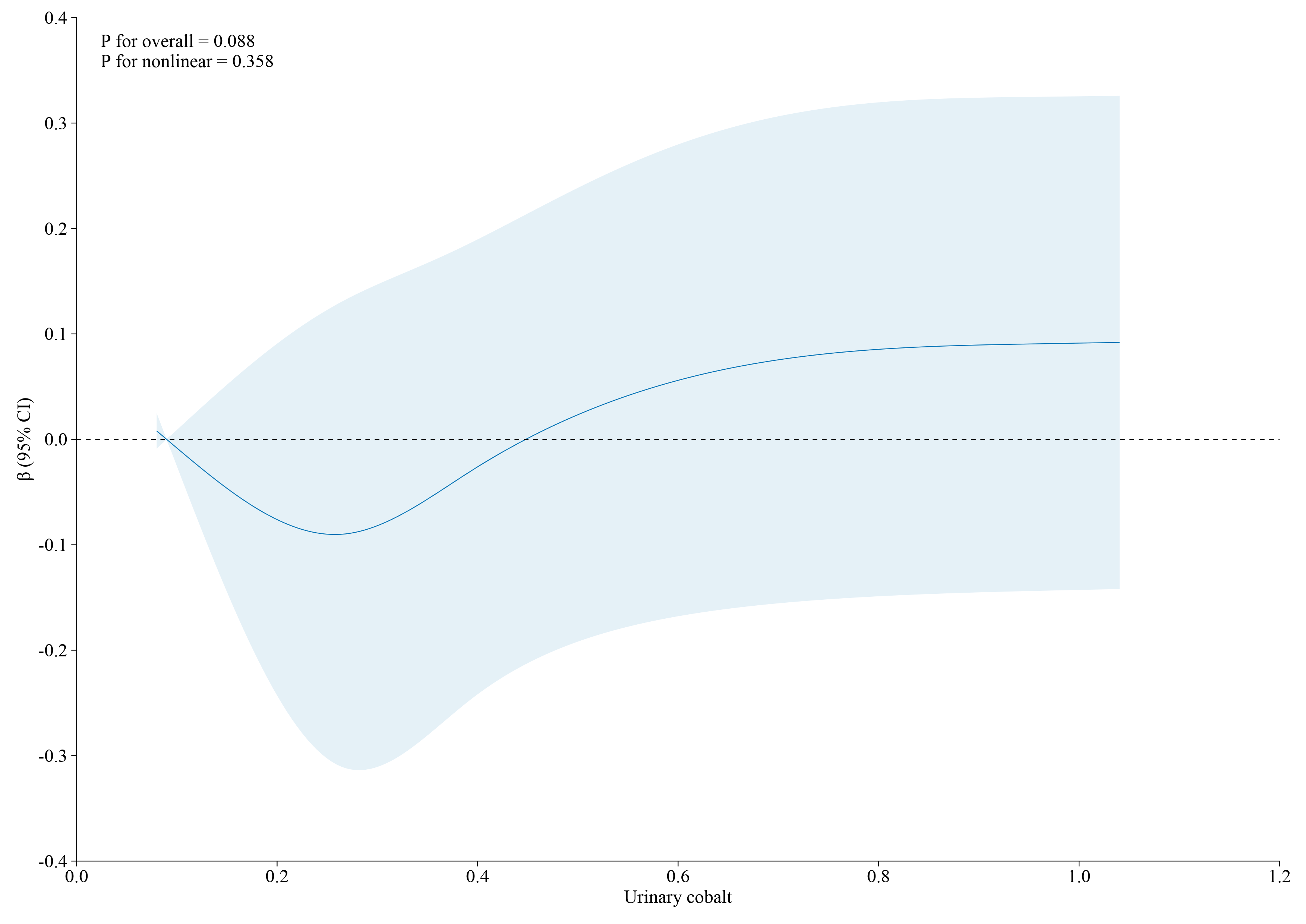

Supplement: SUPPLEMENTARY FIGURE S2 — Restricted cubic spline for association between Be concentration with PSA among non-depression people. Adjusted age, gender, race, educational levels, BMI, family income-poverty ratio level, married status, smoking status, drinking status, diabetes and hypertension. [file Image_2.TIF]

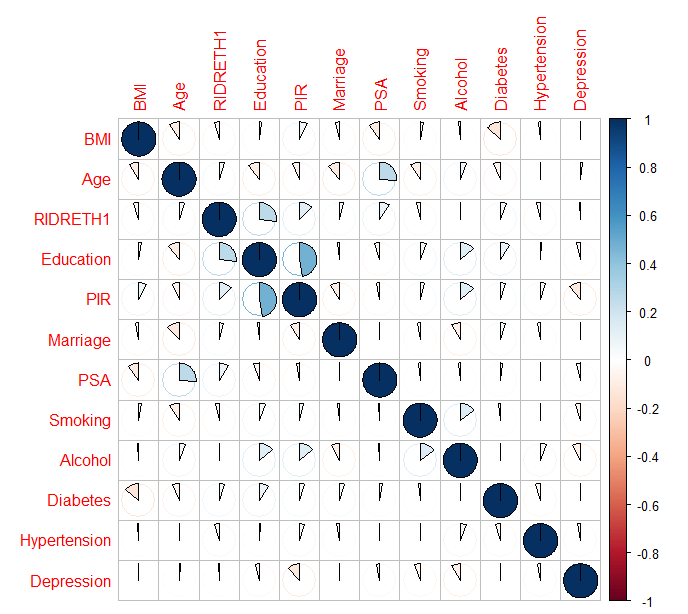

Supplement: SUPPLEMENTARY FIGURE S3 — Spearman correlation matrix between included covariates and PSA. [file Image_3.TIFF]
