# Supplementary material for: Association between urinary metals and prostate-specific antigen in aging population with depression: a cross-sectional study
Source: Front Public Health. 2024 May 23;12:1401072. doi: 10.3389/fpubh.2024.1401072 (PMC11153824; doi:10.3389/fpubh.2024.1401072)
Supplement: Supplementary file 4 [file Table_1.DOCX]

**Table S1.** Subgroup analysis of association between Prostate-Specific Antigen and urinary beryllium in NHANES 2001-2010.

|  | age<60 | age≥60 | BMI<25 | 25≤BMI<30 | BMI≥30 |
| --- | --- | --- | --- | --- | --- |
| **Urinary beryllium** | Reference | Reference | Reference | Reference | Reference |
| Q2 | **0.03(0.01, 0.08)*** | **0.05(0.03, 0.12)*** | - | - | - |
| Q3 | 0.05(-0.02, 0.11) | **0.07(0.05, 0.17)*** | 0(0,0) | **0.01(0.01,1.45)*** | 0(0,0) |
| Q4 | 0.04(-0.03, 0.13) | **0.09(0.05, 0.21)*** | 0.58(-1.7,2.37) | **0.03(0.02,1.27)*** | **0.2(0.08,0.71)*** |
| **p trend** | 0.02 | **-0.15*** | 0.04 | 0.09 | 0.47 |

*P<0.05, Model = adjusted for urine creatinine, ex, age (years, continuous), age squared, education (less than high school, high school graduate, some college and above), race (non-Hispanic white, non-Hispanic black, Mexican American, other), self-reported alcohol status (Yes and No) and self-reported smoking status (Current, Past and Never), BMI, self-reported hypertension (Yes and No) and self-reported diabetes (Yes and No).
