# Supplementary material for: Association between urinary metals and prostate-specific antigen in aging population with depression: a cross-sectional study
Source: Front Public Health. 2024 May 23;12:1401072. doi: 10.3389/fpubh.2024.1401072 (PMC11153824; doi:10.3389/fpubh.2024.1401072)
Supplement: Supplementary file 5 [file Table_2.DOCX]

**Table S2.** Association between Prostate-Specific Antigen and urine metals among non-depression participants.

|  | **Model1 β(95%CI)** | **Model2 β(95%CI)** | **Model3 β(95%CI)** |
| --- | --- | --- | --- |
| **Urinary barium** | Reference | Reference | Reference |
| Q2 | -0.05(-0.17, 0.07) | -0.02(-0.13, 0.09) | -0.04(-0.15, 0.07) |
| Q3 | -0.02(-0.15, 0.1) | 0.05(-0.07, 0.17) | 0.02(-0.1, 0.14) |
| Q4 | -0.03(-0.16, 0.11) | 0.02(-0.11, 0.14) | 0(-0.12, 0.12) |
| **p trend** | 0.86 | 0.57 | 0.73 |
| **Urinary beryllium** | Reference | Reference | Reference |
| Q2 | 0.45(0.33, 0.57) | 0.34(0.23, 0.45) | -0.04(-0.15, 0.07) |
| Q3 | 0.37(0.24, 0.51) | 0.29(0.16, 0.42) | 0.02(-0.1, 0.14) |
| Q4 | 0(0, 0) | 0(0, 0) | 0(-0.12, 0.12) |
| **p trend** | 0.26 | 0.46 | 0.58 |
| **Urinary cadmium** | Reference | Reference | Reference |
| Q2 | 0(-0.1, 0.11) | -0.03(-0.13, 0.06) | -0.04(-0.14, 0.05) |
| Q3 | 0.1(-0.02, 0.23) | 0.02(-0.1, 0.14) | 0.01(-0.11, 0.13) |
| Q4 | 0.17(0.04, 0.29) | 0.01(-0.12, 0.14) | -0.03(-0.16, 0.11) |
| **p trend** | 0.00 | 0.70 | 0.96 |
| **Urinary cobalt** | Reference | Reference | Reference |
| Q2 | 0.02(-0.1, 0.14) | **0.34(0.23, 0.46)*** | -0.02(-0.12, 0.08) |
| Q3 | 0.07(-0.06, 0.21) | **0.29(0.16, 0.44)*** | 0.04(-0.08, 0.17) |
| Q4 | 0.04(-0.1, 0.19) | 0(0, 0) | -0.02(-0.15, 0.12) |
| **p trend** | 0.50 | 0.99 | 0.91 |
| **Urinary cesium** | Reference | Reference | Reference |
| Q2 | 0.04(-0.09, 0.17) | 0.03(-0.09, 0.14) | 0(-0.12, 0.11) |
| Q3 | 0.03(-0.1, 0.16) | 0.06(-0.06, 0.18) | 0.04(-0.08, 0.15) |
| Q4 | -0.04(-0.18, 0.11) | -0.01(-0.16, 0.13) | -0.05(-0.18, 0.09) |
| **p trend** | 0.52 | 0.90 | 0.60 |
| **Urinary molybdenum** | Reference | Reference | Reference |
| Q2 | 0.09(-0.03, 0.21) | 0.05(-0.06, 0.15) | 0.04(-0.06, 0.15) |
| Q3 | 0.05(-0.09, 0.18) | 0.03(-0.09, 0.16) | 0.03(-0.1, 0.15) |
| Q4 | 0.05(-0.08, 0.19) | 0.01(-0.12, 0.14) | -0.01(-0.13, 0.12) |
| **p trend** | 0.64 | 0.98 | 0.81 |
| **Urinary lead** | Reference | Reference | Reference |
| Q2 | 0(-0.12, 0.12) | -0.05(-0.16, 0.06) | -0.06(-0.17, 0.05) |
| Q3 | 0(-0.12, 0.13) | -0.02(-0.14, 0.09) | -0.04(-0.15, 0.08) |
| Q4 | 0(-0.13, 0.13) | -0.1(-0.23, 0.02) | -0.11(-0.24, 0.02) |
| **p trend** | 0.96 | 0.17 | 0.15 |
| **Urinary antimony** | Reference | Reference | Reference |
| Q2 | -0.1(-0.22, 0.01) | -0.09(-0.19, 0.02) | -0.09(-0.19, 0.01) |
| Q3 | -0.12(-0.25, 0.01) | -0.07(-0.19, 0.05) | -0.07(-0.18, 0.05) |
| Q4 | -0.17(-0.3, -0.04) | -0.13(-0.25, 0) | -0.12(-0.24, 0) |
| **p trend** | 0.11 | 0.06 | 0.07 |
| **Urinary thallium** | Reference | Reference | Reference |
| Q2 | 0.04(-0.09, 0.17) | 0.02(-0.1, 0.14) | 0.02(-0.1, 0.14) |
| Q3 | -0.08(-0.22, 0.05) | -0.04(-0.17, 0.09) | -0.05(-0.17, 0.08) |
| Q4 | -0.09(-0.24, 0.07) | -0.01(-0.16, 0.13) | -0.02(-0.16, 0.12) |
| **p trend** | 0.11 | 0.7 | 0.6 |

*P<0.05, Model 1= adjusted for urine creatinine; Model 2 = as Model 1 plus adjusted for sex, age (years, continuous), age squared, education (less than high school, high school graduate, some college and above), race (non-Hispanic white, non-Hispanic black, Mexican American, other), self-reported alcohol status (Yes and No) and self-reported smoking status (Current, Past and Never); Model 3 = Model 2 plus adjusted for BMI, self-reported hypertension (Yes and No) and self-reported diabetes (Yes and No).
